# The Urotensin System Is Up-Regulated in the Pre-Hypertensive Spontaneously Hypertensive Rat

**DOI:** 10.1371/journal.pone.0083317

**Published:** 2013-12-05

**Authors:** Ellen J. Forty, Nick Ashton

**Affiliations:** Faculty of Life Sciences, University of Manchester, Manchester, United Kingdom; University Medical Center Utrecht, Netherlands

## Abstract

Urotensin II (UII) concentrations are raised both in humans with hypertension and in spontaneously hypertensive rats (SHR). Since the urotensin system acts to regulate glomerular filtration in the kidney it may play a greater role in the pre-hypertensive SHR in which renal dysfunction is known to precede the onset of severe hypertension. This study aimed to determine the renal actions and expression of the urotensin system in the young SHR. Intravenous rat UII (6 pmol. min^-1^. 100 g body weight^-1^) had no significant effect on GFR; however urotensin-related peptide (URP) reduced GFR (P<0.05) in 4-5 week old SHR. Administration of the UT antagonist SB-706375 evoked marked increases in GFR (baseline 0.38 ± 0.07 vs antagonist 0.76 ± 0.05 ml. min^-1^. 100 g body weight^-1^, P<0.05), urine flow and sodium excretion (baseline 2.5 ± 0.4 vs antagonist 9.1 ± 2.1 µmol. min^-1^. 100 g body weight^-1^, P<0.05) in the SHR. Normotensive Wistar-Kyoto rats showed little response to UT antagonism. Quantitative RT-PCR showed that neither UII nor UT mRNA expression differed between the kidneys of young SHR and WKY rats; however expression of URP was 4-fold higher in the SHR kidney. Renal transcriptional up-regulation indicates that URP is the major UT ligand in young SHR and WKY rats. Enhanced tonic UT activation may contribute to known renal dysfunction in pre-hypertensive SHR.

## Introduction

Urotensin II (UII) and its receptor, UT, are expressed abundantly in the kidneys of humans [1], rats [2] and other mammals [3]. Urotensin-related peptide (URP), an homologous alternative ligand with a similar affinity and potency to UII at UT [4], is also expressed in both human [4] and rat [2] kidneys. Collectively, the urotensin system contributes to the regulation of renal function primarily through tonic modulation of glomerular filtration rate (GFR) [2,5]. UT activation leads to a reduction in urinary sodium and water loss, as a result of a decrease in GFR. In addition, UII has a direct effect on the renal tubule, where it acts to inhibit sodium reabsorption [5]. The actions of UII are dose-related, with sodium loss enhanced at low doses and sodium retention stimulated at higher doses [5]. Thus up-regulation of the renal urotensin system is likely to lead to sodium retention and hence an increase in systemic blood pressure.

Several studies have shown a positive correlation between elevated UII concentrations in circulating plasma [6,7], cerebrospinal fluid [8] or urine [1] and human hypertension. Furthermore, a genetic polymorphism of the UII gene allele frequency has been associated with essential hypertension in a Han Chinese population [9]. However there have also been some contradictory reports in which UII concentrations were inversely related to blood pressure [10] and a cardioprotective role has been proposed for UII in patients with end stage renal disease [11,12]. Collectively, these findings suggest that the urotensin system plays a role in cardiovascular disease; however that role may be protective or deleterious depending on the particular pathology.

In the adult spontaneously hypertensive rat (SHR) urotensin system expression is elevated in the heart, aorta and kidneys [2,13,14]. The plasma UII concentration is 2-fold higher in SHR compared with control Wistar-Kyoto (WKY) rats [2,14] and both pressor [15] and hypotensive [14] responses to UII are exaggerated in the adult SHR compared with WKY rats. Exogenous UII infusion results in an enhanced anti-natriuresis in the SHR [13,14] which is driven by a reduction in GFR [13]. These data reflect the observations made in humans with established cardiovascular disease. What is unclear is the role played by the urotensin system during the development of hypertension.

Blood pressure in the SHR increases gradually from 4-6 weeks of age, then rapidly through to 10 weeks of age, before reaching a plateau [16]. The period between 4-6 weeks of age is considered to be the ‘pre-hypertensive’ phase during which mean arterial blood pressure is higher than in WKY rats but has yet to reach the full hypertensive phenotype (SBP >200 mmHg). During this pre-hypertensive phase transplantation studies have shown that renal abnormalities are responsible for the development of hypertension in the SHR [17]. Pre-hypertensive SHRs have lower GFRs [18] and a positive cumulative sodium and water balance compared with age-matched WKY rats [16]. The subsequent expansion of extracellular fluid volume leads to an increase in systemic blood pressure. As the SHR matures renal function is normalised at the expense of elevated blood pressure [18].

The mechanisms responsible for the initial reduction in GFR in the young SHR are not understood fully. We have reported recently that the urotensin system exerts a tonic influence on GFR and sodium and water excretion in 4 week-old Sprague Dawley rats [19]. Consequently, in view of the enhanced renal sensitivity to UII that we observed in adult SHR [13], we hypothesise that the urotensin system contributes to the lower GFR of the young, pre-hypertensive SHR. Accordingly, the aim of this study was to determine the influence of the urotensin system on renal function in the young SHR. We demonstrate enhanced GFR-mediated responses to UT antagonism in pre-hypertensive SHR compared to age-matched WKY rats and identify URP as the major transcript ligand in young rats associated with a pre-hypertensive phenotype. 

## Methods

All experiments were performed in accordance with the UK Animals (Scientific Procedures) Act 1986 and received local ethical approval from the University of Manchester. All chemicals and reagents are from Sigma (Poole, Dorset, UK) unless stated otherwise.

### Animals

Male 3-4 week-old spontaneously hypertensive rats (SHRs) and Wistar-Kyoto (WKY) rats were purchased from Harlan UK Ltd (Oxon, UK), and were housed in the Biological Sciences Facility for 1 week before use. All animals were kept in a 12 hour light cycle, with regulated temperature and humidity and *ad libitum* access to food and water.

### Effect of exogenous rat UII administration on renal function

Inactin (thiobutabarbital sodium, 10 mg/100 g body weight i.p.) anaesthetised 4-5 week-old WKY rats and SHRs were surgically prepared for renal clearance measurements [19]. A priming dose of renal clearance markers was administered via a jugular catheter (2 μCi ^3^H inulin [Perkin-Elmer, Monza, Italy] and 12 mg para-aminohippuric acid [PAH] in 0.2 ml 0.154 mol/L NaCl). Following a 2 h equilibration period under continuous infusion of 0.154 mol/L NaCl and clearance markers (^3^H inulin 0.6 μCi/ml and PAH 1.25 mg/ml) at 40 μl/min, urine collection via a bladder catheter began at 15 min intervals for an initial 30 min control period. After this control period animals were selected randomly to receive either vehicle (0.154 mol/L NaCl at 40 μl/min, n = 6 per strain), rat UII (rUII, Peptide Institute, Osaka, Japan) at 6 pmol. min^-1^. 100 g bwt^-1^ (n = 7 per strain) or URP (Phoenix Pharmaceuticals, CA, USA) at 6 pmol. min^-1^. 100 g bwt^-1^ (n = 5 per strain) for 1 h. This dose of UII was selected as being physiologically relevant with a minimal effect on the systemic and renal vasculature [2]. Blood pressure was recorded throughout the renal clearance experiment via a catheter implanted in the left carotid artery (Powerlab 800/s, ADInstruments, Hastings, East Sussex, UK). Blood samples were taken (0.4 ml) from the carotid artery catheter midway through the control and experimental periods and replaced with an equal volume of 0.154 mol/L NaCl; haematocrit was measured at the end of the experiment for the subsequent calculation of effective renal blood flow. At the end of the experiment urine flow rate was measured gravimetrically and urine and plasma samples were analysed for ^3^H inulin activity (1900CA Tri-Carb Liquid Scintillation Analyser, Canberra Industries, Meriden, CT), PAH (standard colorimetric assay) and Na^+^ concentrations (atomic absorption spectroscopy, Solaar S Series, Thermo Elemental, Unicam, Cambridge, UK). Animals were killed humanely by cervical dislocation at the end of the experiment. 

### Effect of UT antagonist administration on renal function

A separate group of 4-5 week-old WKY rats and SHRs were surgically prepared for renal clearance measurements as described above. Following the 2 h equilibration period urine was collected every 15 min for an initial 45 min control period. After the control period animals received either vehicle (0.154 mol/L NaCl, n = 5 per strain) or the non-peptide UT antagonist SB-706375 ((2-bromo-4,5-dimethoxy-N-[3-(R)-1-methyl-pyrrolidin-3-yloxy)-4-trifluromethyl-phenyl]-benzenesulphonamide HCl);[20]) at 0.01 mg. kg^-1^. min^-1^ (WKY n = 6, SHR n = 8) for 75 min. We have used this dose of SB-706375, which is full UT antagonist [20], previously to provoke a renal response in the 4 week-old Sprague-Dawley rat [19]. Urine and plasma samples were analysed as described above.

### Quantitative RT-PCR

Quantitative real-time PCR for UII, URP and UT mRNA expression was performed on 4-5 week-old kidneys from WKY rats (n = 5-7) and SHRs (n = 5-8) killed by cervical dislocation. Kidneys were subdivided into cortex and medulla and total RNA was extracted from 50-100 mg tissue using TRIzol reagent (Invitrogen, Paisley, UK). First-strand cDNA was synthesised from 1 μg of DNAse-treated RNA using SuperScript II reverse transcriptase (Invitrogen) and random primers (Roche Diagnostics, Mannheim, Germany). 

Primers and Taqman^Tm^ probes (Eurogentec, Southampton, UK) for rat *Uts2* and housekeeping genes *Gapdh* and *Actb* (primer/probe sequences Table 1) were used with reagents from a qPCR Core kit for probes (with ROX, Eurogentec) to quantify UII mRNA expression in WKY rat and SHR cDNA samples. *Uts2* was normalised against both *Gapdh* and *Actb*. Custom-designed real-time PCR assay kits with PerfectProbe^Tm^ (Table 1) from Primer Design (Southampton, UK) for *Uts2d* and *Uts2r* genes were used to quantify URP and UT mRNA expressions in cDNA samples. A geNorm^Tm^ Housekeeping Gene Selection kit with PerfectProbe^Tm^ (Primer Design) identified *18*S, *Atpb5* and *Top1* as housekeeping genes that were expressed stably across the cDNA samples from 4-5 week-old WKY rat and SHR kidneys, hence *Uts2d* and *Uts2* were normalised against all three housekeeping genes. PerfectProbes^Tm^ were used with reagents from Precision-R^Tm^ 2x qPCR Mastermix (Primer Design) to amplify the relevant mRNAs. Negative controls for both types of assay consisted of replacing cDNA with water, or omitting the primer/probe mix, whilst a BioBank^Tm^ cDNA kit for rat (Primer Design) was used for positive controls on each plate. Triplicate wells of cDNA were amplified on 96-well plates using an ABI PRISM 7300 detector (Applied Biosystems, Foster City, CA, USA). Relative quantification of UII, URP and UT mRNA was calculated using the 2^-ΔΔCT^ method with normalisation to respective housekeeping genes.

**Table 1:** Primers and probe sequences used for quantitative RT-PCR.

**Gene**	**Accession number**	**Primer/probe**	**Position**	**Sequence**
***Uts2***	NM_019160	Forward primer	294	GGCTCTCACTGGGCAAGATTC
		Reverse primer	367	GTTGCTTACGTTGTTTCCTGGTT
		Probe	317	ACACTGTACTGAGCCGTCTTTTGGCGA
***Gapdh***	NM_017008	Forward primer	895	CTACACTGAGGACCAGGTTGTC
		Reverse primer	961	CTGTGACTTCAACAGCA
		Probe	919	CATCAAAGGTGGAAGAATGG
***Actb***	NM_031144	Forward primer	931	GACAGGATGCAGAAGGAGATTACTG
		Reverse primer	1031	GAGCCACCAATCCACACAGA
		Probe	969	CACCATGAAGATCAAGATCATTGCTCCTCCT
***Uts2d***	NM_198133	Forward primer	653	ATGGAACTGAGAACTGCGTATC
		Reverse primer	733	AGACTTCAGGCGAGCATACA
***Uts2r***	NM_020537	Forward primer	343	GACTGGCACTTTGGAGATGT
		Reverse primer	443	CGTTCGCTGCTCATTATGGT

Note: geNorm^TM^ house-keeping gene sequences are not publicly available

### Immunohistochemistry

Kidneys from inactin (thiobutabarbital sodium, 10 mg/100 g body weight i.p.) anaesthetised, male 4-5 week-old WKY rats (n = 4) and SHRs (n = 3) were flushed with PBS and then perfused with 4% PFA before wax embedding. Urotensin peptides (UII and/or URP) were localised in 5 μm sections using a polyclonal rabbit anti-flounder UII antibody (1:200 dilution, raised against the conserved amino-acid sequence Cys-Phe-Trp-Lys-Tyr-Cys by Dr P. Ingleton, University of Sheffield, UK [21]), followed by application of a secondary goat anti-rabbit antibody (1:100, DakoCytomation, Cambridgeshire, UK). A polyclonal goat anti-rat UT receptor antibody (1:100 dilution, Santa Cruz Biotechnology, CA, USA), followed by donkey anti-goat (1:300, DakoCytomation) secondary antibody were used to localise UT protein in kidney sections. Diaminobenzidine (SIGMAFAST™ 3,3′-diaminobenzidine tablets) was used to develop antibody-HRP conjugate. Negative controls consisted of omitting primary antibody. 

### Statistical analyses

**Figure 1:**
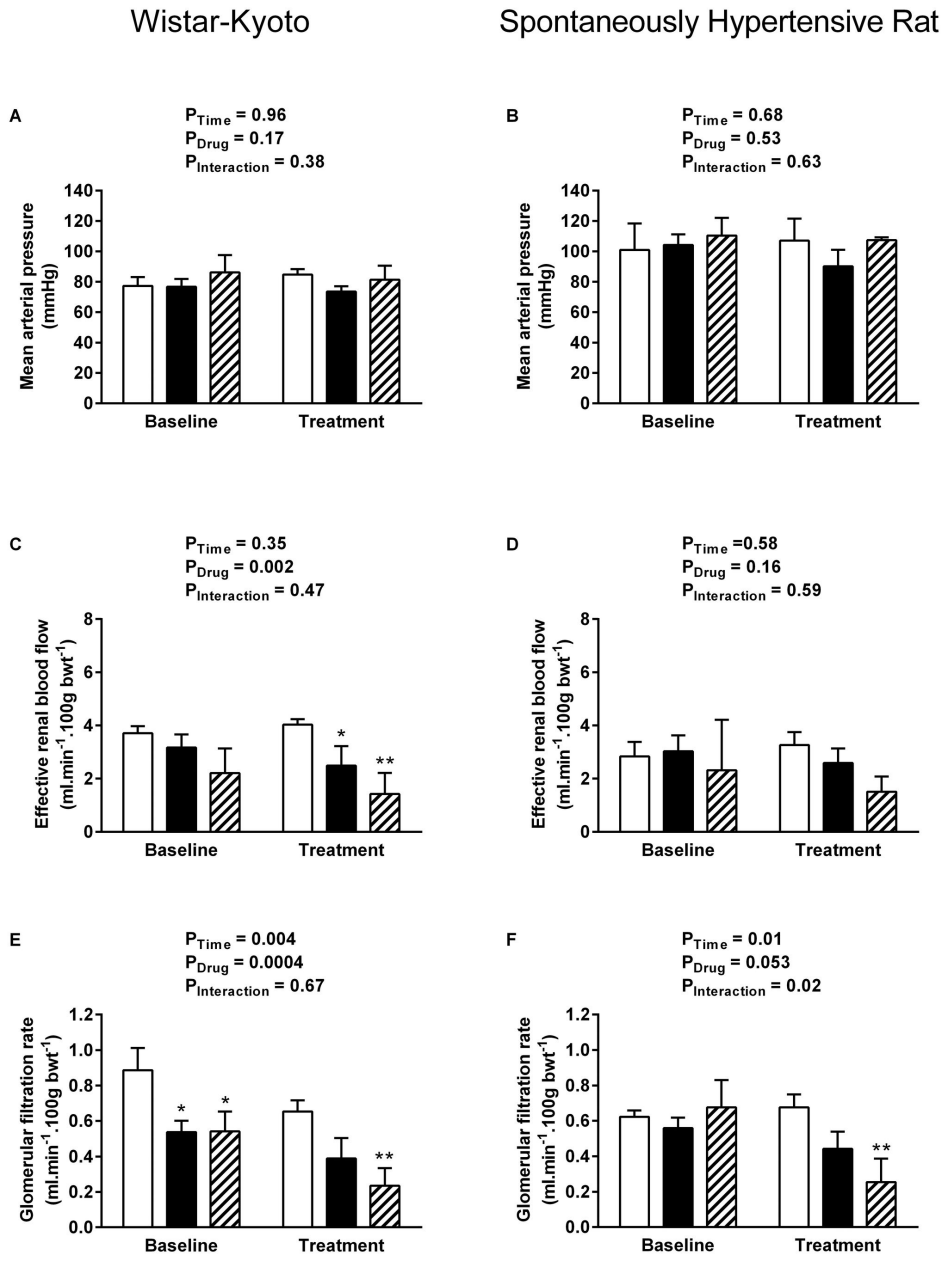
Systemic and renal haemodynamics during rUII and URP infusion. Effect of exogenous rUII (6 pmol. min^-1^. 100 g bwt^-1^, solid bars, n = 7 per strain), URP (6 pmol. min^-1^. 100 g bwt^-1^, hatched bars, n = 5 per strain) or vehicle (0.154 mol/L NaCl, open bars, n = 6 per strain) infusion on mean arterial pressure (A-B), effective renal blood flow (C-D) and glomerular filtration rate (E-F) in anaesthetised 4-5 week-old WKY rats (left column) and pre-hypertensive SHRs (right column). Data shown are mean ± SEM for the baseline control period and the final 15 mins of the treatment period when effects were maximal. * P < 0.05, ** P < 0.01 compared with vehicle-treated rats, Dunnett’s test.

**Figure 2:**
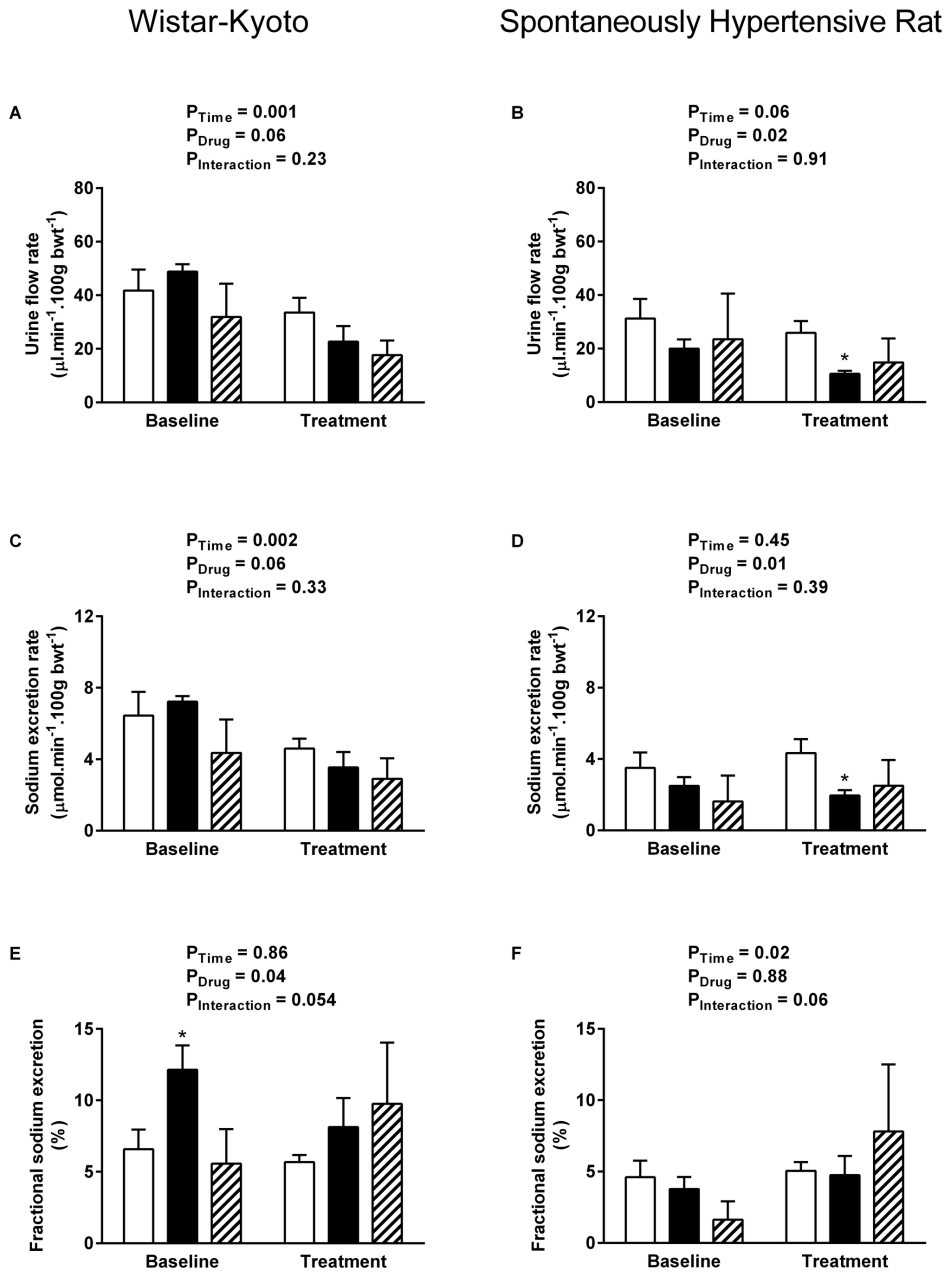
Renal water and sodium handling during rUII and URP infusion. Effect of exogenous rUII (6 pmol. min^-1^. 100 g bwt^-1^, solid bars, n = 7 per strain), URP (6 pmol. min^-1^. 100 g bwt^-1^, hatched bars, n = 5 per strain) or vehicle (0.154 mol/L NaCl, open bars, n = 6 per strain) infusion on urine flow rate (A-B), sodium excretion rate (C-D) and fractional sodium excretion (E-F) in anaesthetised 4-5 week-old WKY rats (left column) and pre-hypertensive SHRs (right column). Data shown are mean ± SEM for the baseline control period and the final 15 mins of the treatment period when effects were maximal. * P < 0.05 compared with vehicle-treated rats, Dunnett’s test.

**Figure 3:**
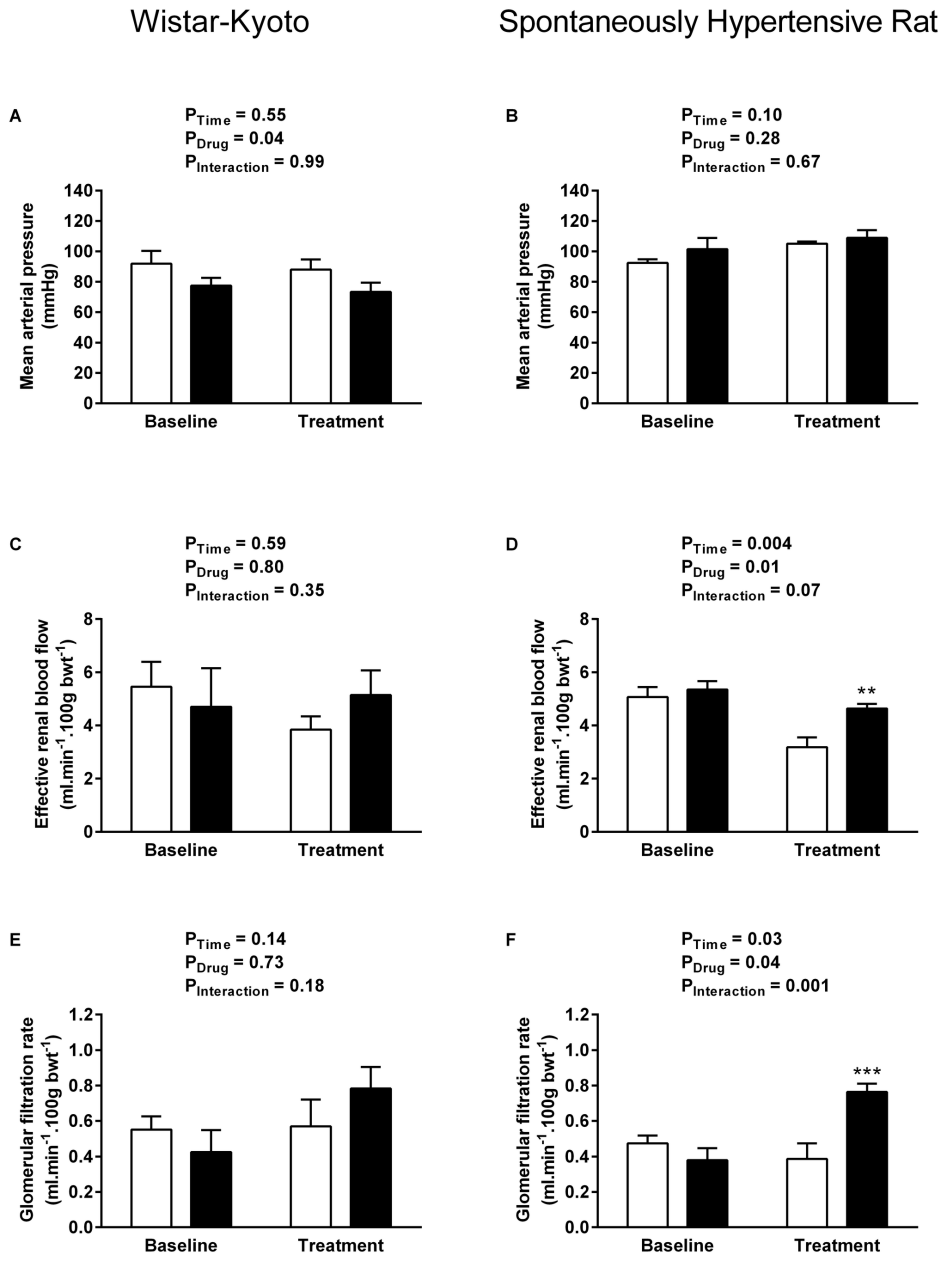
Systemic and renal haemodynamics during UT antagonism. Effect of UT antagonist SB-706375 (0.01 mg. kg^-1^. min^-1^, solid bars, WKY n = 6, SHR n = 8) or vehicle (0.154 mol/L NaCl, open bars, n = 5 per strain) infusion on mean arterial pressure (A-B), effective renal blood flow (C-D) and glomerular filtration rate (E-F) in anaesthetised 4-5 week-old WKY rats (left column) and pre-hypertensive SHRs (right column). Data shown are mean ± SEM for the baseline control period and the final 15 mins of the treatment period when effects were maximal. ** P < 0.01, *** P < 0.001 compared with vehicle-treated rats, Sidak’s test.

**Figure 4:**
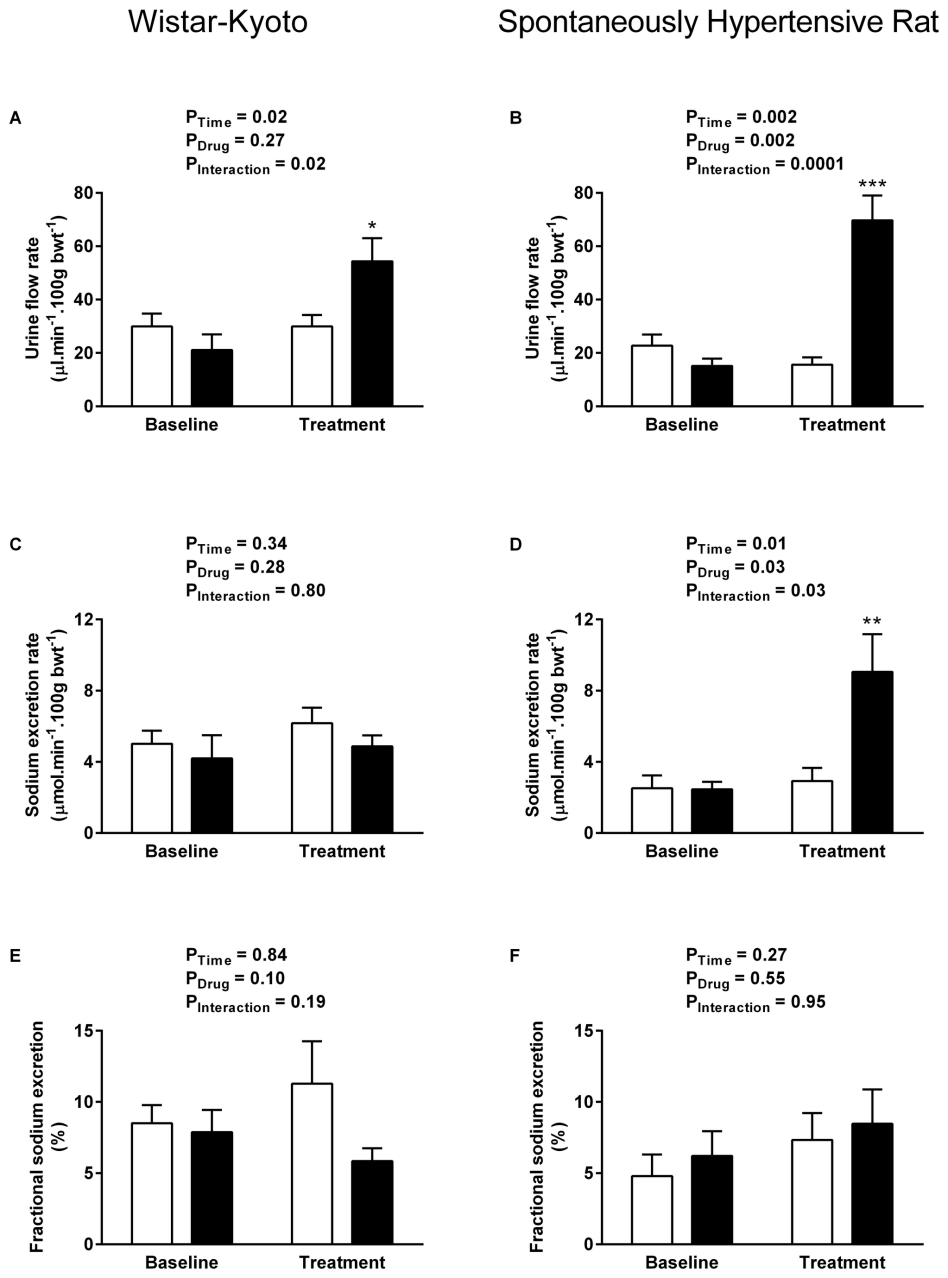
Renal water and sodium handling during UT antagonism. Effect of UT antagonist SB-706375 (0.01 mg. kg^-1^. min^-1^, solid bars, WKY n = 6, SHR n = 8) or vehicle (0.154 mol/L NaCl, open bars, n = 5 per strain) infusion on urine flow rate (A-B), sodium excretion rate (C-D) and fractional sodium excretion (E-F) in anaesthetised 4-5 week-old WKY rats (left column) and pre-hypertensive SHRs (right column). Data shown are mean ± SEM for the baseline control period and the final 15 mins of the treatment period when effects were maximal. * P <0.05, ** P < 0.01, *** P < 0.001 compared with vehicle-treated rats, Sidak’s test.

**Figure 5:**
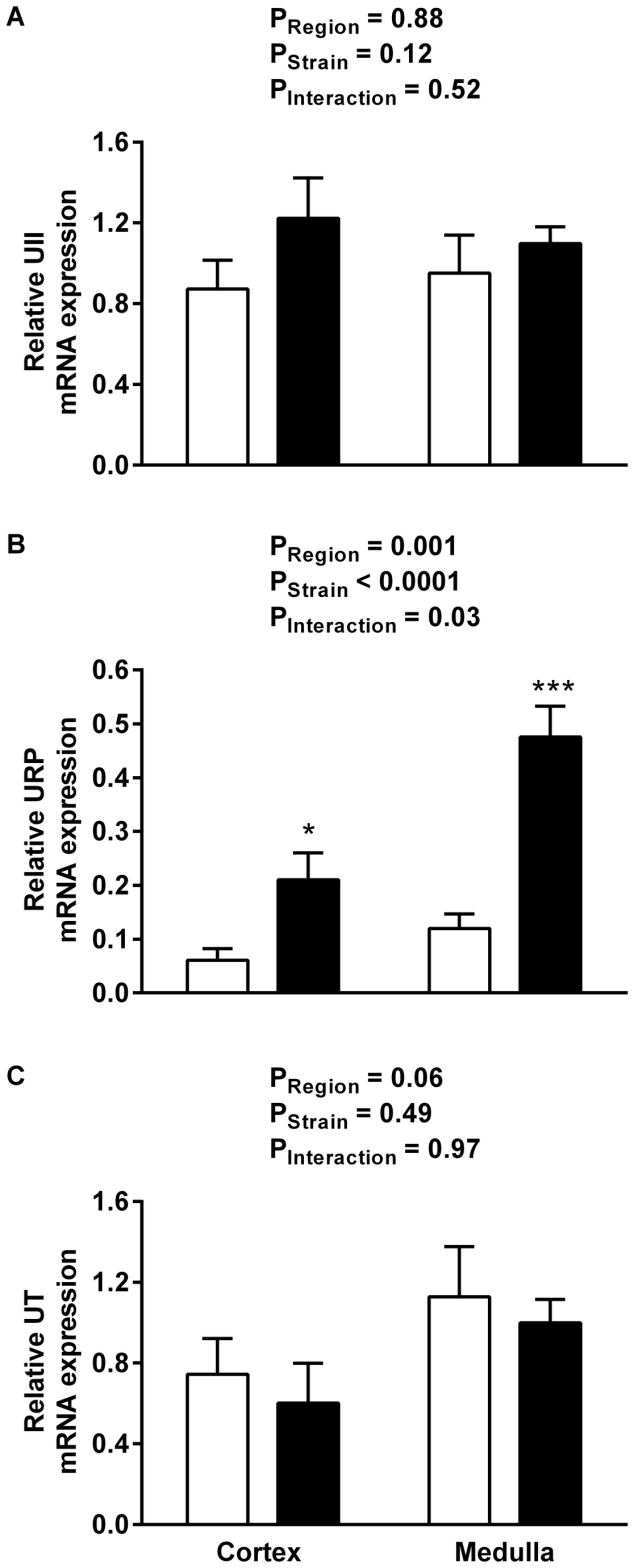
Renal urotensin system relative mRNA expression. Relative UII (A), URP (B) and UT (C) mRNA expression in the renal cortex and medulla of WKY rats (open bars) and SHRs (soild bars) at 4-5 weeks of age. Data shown are mean ± SEM (n = 7-8). * P <0.05, *** P < 0.001 compared with WKY rats, Sidak’s test.

All data are presented as mean ± SEM and were analysed by two-way ANOVA using SPSS for Windows (v15, SPSS UK, Surrey, UK). Multiple comparison post hoc tests (Dunnett’s test Figures 1 & 2; Sidak’s test Figures 3-5) were performed to identify differences between specific groups. Significance was ascribed at the 5% level. 

## Results

### GFR is reduced by exogenous URP but not by rUII in the pre-hypertensive SHR

In accord with previous studies [16] baseline mean arterial pressure (MAP) was significantly higher whereas urine flow rate (UV), sodium excretion rate (U_Na_V) and fractional sodium excretion (FE_Na_) were significantly lower in young SHR (Table 2). Baseline effective renal blood flow (ERBF) and glomerular filtration rate (GFR) did not differ between strains under the experimental conditions employed. 

**Table 2:** Baseline measurements taken during control 0.154 mol/L NaCl infusion before rUII or URP infusion in anaesthetised 4-5 week-old SHR and WKY rats.

**Parameter**	**WKY**			**SHR**			**P value**
	**Vehicle**	**rUII**	**URP**	**Vehicle**	**rUII**	**URP**	
	**(n = 6)**	**(n = 7)**	**(n = 5)**	**(n = 6)**	**(n = 7)**	**(n = 5)**	
**MAP (mmHg)**	77 ± 6	78 ± 6	86 ± 5	104 ± 14	104 ± 7	110 ± 5	<0.001
**ERBF (ml. min^-1^. 100 g bwt^-1^)**	3.7 ± 0.3	3.3 ± 0.5	2.2 ± 0.4	2.8 ± 0.4	3.0 ± 0.6	2.3 ± 0.9	NS
**GFR (ml. min^-1^. 100 g bwt^-1^)**	0.9 ± 0.1	0.5 ± 0.1	0.5 ± 0.1	0.6 ± 0.03	0.6 ± 0.1	0.7 ± 0.1	NS
**UV (μl. min^-1^. 100 g bwt^-1^)**	39.0 ± 7.1	44.0 ± 6.1	31.9 ± 5.5	31.2 ± 7.1	21.8 ± 3.5	23.5 ± 7.6	<0.01
**U_Na_V (μmol. min^-1^. 100 g bwt^-1^)**	6.4 ± 1.3	6.4 ± 0.8	4.4 ± 0.8	3.1 ± 0.8	2.8 ± 0.5	1.6 ± 0.6	<0.001
**FE_Na_ (%)**	6.6 ± 1.4	11.2 ± 1.9	5.6 ± 1.1	3.9 ± 1.1	4.3 ± 0.8	1.6 ± 0.6	<0.001

Data shown are mean ± SEM

MAP: mean arterial pressure, ERBF: effective renal blood flow, GFR: glomerular filtration rate, UV: urine flow rate, U_Na_V: sodium excretion rate, FE_Na_: fractional sodium excretion, NS: not significant.

MAP remained stable in all groups of animals and was unaffected by infusion of either exogenous rUII or URP at 6 pmol.min^-1^.100 g bwt^-1^ (Figure 1A-B). WKY rats responded to both rUII (Figure 1C P = 0.049) and URP (Figure 1C P = 0.002) infusion with significant reductions in effective renal blood flow (ERBF). In contrast ERBF was unaffected by rUII or URP in SHR (Figure 1D). Rat UII tended to lower GFR in both WKY (Figure 1E P = 0.06) and SHR (Figure 1F P = 0.052); whereas URP infusion resulted in significant reductions in both strains (WKY P = 0.006, SHR P = 0.001) compared with vehicle-treated time controls. Neither rat UII nor URP infusion had an effect on urine flow rate (Figure 2A), sodium excretion rate (Figure 2C) or fractional sodium excretion (Figure 2E) in the WKY animals. In contrast young SHR responded to rUII with significant reductions in urine flow rate (Figure 1B, P = 0.05) and sodium excretion rate (Figure 1D, P = 0.02), without a change in fractional sodium excretion (Figure 2F). Urine flow and sodium excretion rates tended to fall during URP infusion in the SHR; however these changes did not reach statistical significance.

### UT antagonist infusion reveals tonic influence of endogenous urotensin peptides on renal haemodynamics in the pre-hypertensive SHR

As UT receptor occupancy is high under basal conditions due to the high affinity and pseudo-irreversible binding properties of UII [22], we used the UT antagonist SB-706375 in order to reveal the tonic influence of endogenous urotensin peptides (UII and URP) in a naive group of young SHR and WKY rats. Baseline measurements were similar to those in the previous experiment (Table 3); UT antagonist infusion had no effect on MAP in the SHR (Figure 3B), but lowered pressure in the WKY rats (Figure 3A, P = 0.04).

**Table 3:** Baseline measurements taken during control 0.154 mol/L NaCl infusion before UT antagonist infusion in anaesthetised 4-5 week-old SHR and WKY rats.

**Parameter**	**WKY**		**SHR**		**P value**
	**Vehicle**	**UT antagonist**	**Vehicle**	**UT antagonist**	
	**(n = 5)**	**(n = 6)**	**(n = 5)**	**(n = 8)**	
**MAP (mmHg)**	90 ± 8	76 ± 5	92 ± 2	100 ± 7	0.059
**ERBF (ml. min^-1^. 100 g bwt^-1^)**	5.8 ± 0.8	5.8 ± 0.9	6.1 ± 1.0	6.3 ± 0.4	NS
**GFR (ml. min^-1^. 100 g bwt^-1^)**	0.6 ± 0.1	0.5 ± 0.1	0.6 ± 0.1	0.5 ± 0.1	NS
**UV (μl. min^-1^. 100 g bwt^-1^)**	29.3 ± 4.5	21.7 ± 5.3	26.1 ± 8.1	13.8 ± 2.7	NS
**U_Na_V (μmol. min^-1^. 100 g bwt^-1^)**	5.0 ± 0.8	3.9 ± 1.0	2.7 ± 0.8	1.7 ± 0.3	<0.01
**FE_Na_ (%)**	7.8 ± 0.9	8.2 ± 1.6	3.9 ± 1.3	4.8 ± 0.4	<0.05

Data shown are mean ± SEM

MAP: mean arterial pressure, ERBF: effective renal blood flow, GFR: glomerular filtration rate, UV: urine flow rate, U_Na_V: sodium excretion rate, FE_Na_: fractional sodium excretion, NS: not significant.

UT antagonist infusion had no apparent effect on any of the measured renal variables in the WKY cohort, with no significant impact on ERBF (Figure 3C), GFR (Figure 3E), urine flow (Figure 4A), sodium excretion (Figure 4C) or fractional excretion of sodium (Figure 4E).

In contrast SB-706375 evoked pronounced changes in the young SHR, resulting in a 2-fold increase in GFR (Figure 3F, P = 0.04); a modest increase in effective renal blood flow was also apparent compared with the vehicle-treated time control (Figure 3D). The subsequent increase in filtered load was associated with a 4-fold increase in urine flow rate (Figure 4B baseline 15.2 ± 2.7 vs antagonist 69.7 ± 9.3 μl. min^-1^. 100 g bwt^-1^, P = 0.002,) and a 3-fold increase in sodium excretion (Figure 4D, baseline 2.5 ± 0.4 vs antagonist 9.1 ± 2.1 μmol. min^-1^. 100 g bwt^-1^, P = 0.03). The natriuresis appears to be due to the increase in GFR rather than an alteration in tubular handling of sodium, since UT antagonist infusion had no effect on fractional excretion of sodium in the young SHR (Figure 4F).

### Increased URP mRNA expression in the young SHR

Quantitative RT-PCR was used to compare regional expression of UII, URP and UT mRNA in the kidneys of SHR and WKY rats. Neither UII (Figure 5A) nor UT mRNA expression (Figure 5C) differed significantly between the strains or across the kidney. In contrast there was a significant increase in the alternative ligand URP mRNA relative expression in the SHR medulla compared with the SHR cortex and both the WKY medulla and cortex (P<0.001, Figure 5B). 

### Renal UII and UT protein localisation does not differ between SHR and WKY rats

**Figure 6:**
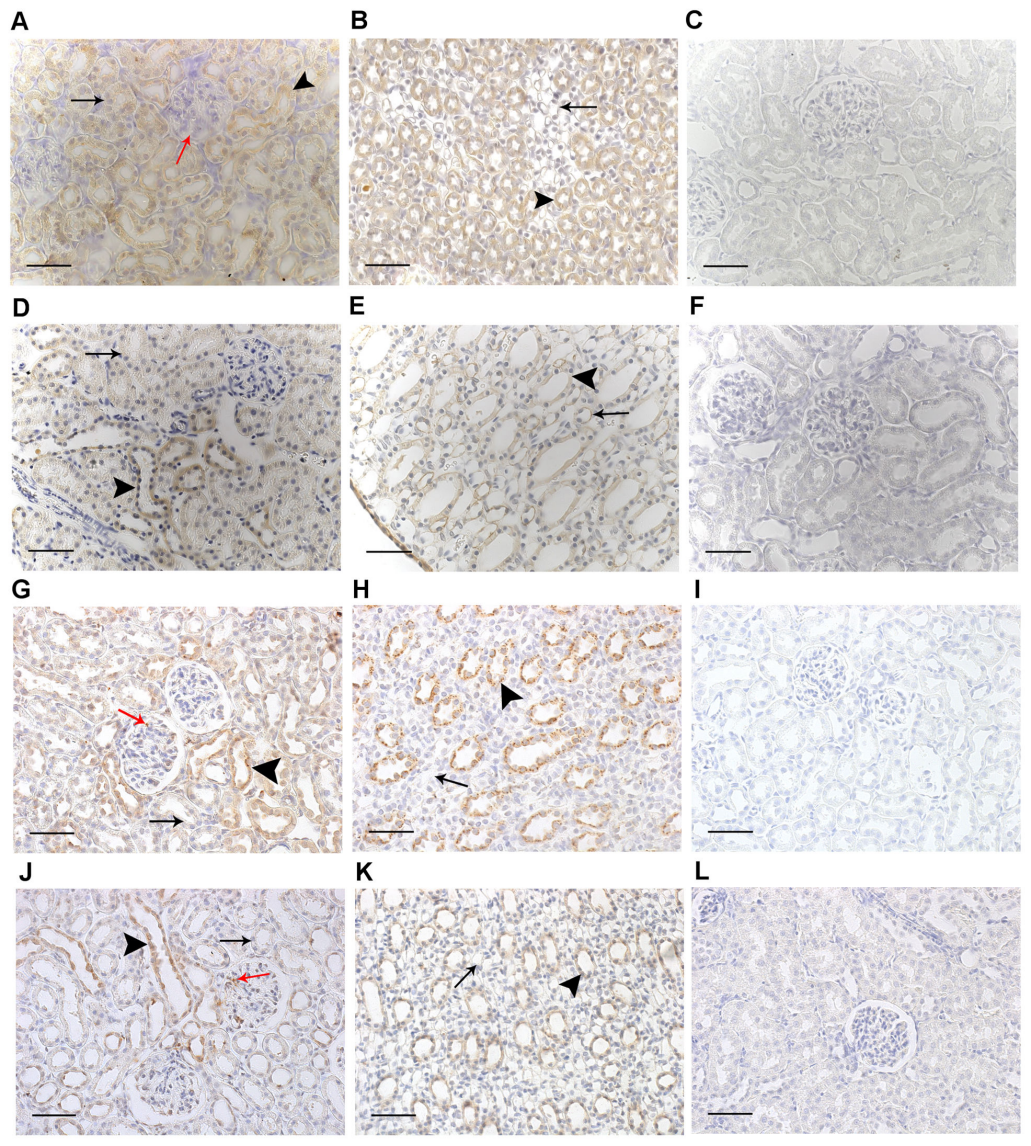
Immunolocalisation of urotensin system proteins in the pre-hypertensive SHR and WKY rat kidney. UII (A-F) and UT (G-L) proteins in the kidneys of 4-5-week-old WKY rats and SHRs. All images are representative and captured at x40 magnification (scale bar 50 µm). Images in the right hand column (C, F, I and L) represent typical negative control sections where primary antibody was omitted. UII-immunoreactivity in both the WKY rat (A) and the SHR (D) cortex was diffuse in distal (arrowheads) and proximal (black arrows) tubules, with no UII-immunoreactivity in glomeruli (red arrow, A). UII-immunoreactivity in both the WKY rat (B) and the SHR (E) medulla was located principally in the collecting ducts (arrowheads), with some immunoreactivity in the loops of Henle (arrows). In both the WKY rat (G) and the SHR (J) cortex UT-immunoreactivity was more intense in the distal (arrowheads) compared to proximal (black arrows) tubules, with UT-immunoreactivity absent in the SHR proximal tubules (black arrow, J); there was also some immunoreactivity in the glomeruli (red arrows). UT-immunoreactivity in both the WJY rat (H) and the SHR (K) medulla was localised to collecting ducts (arrowheads), with little immunoreactivity in the loops of Henle (arrows).

At 4-5 weeks of age UII immunoreactivity (representing cross-reactivity with both UII and URP) was abundant and diffuse, localised to cortical tubules (with more intense immunoreactivity in the distal than proximal tubules, Figure 6A and D) and medullary collecting ducts and loops of Henle (Figure 6B and E) in both strains. Conversely in both strains UT-immunoreactivity was localised primarily to the distal tubules of the cortex, with additional clusters of positive staining in the glomeruli (Figure 6G and J), as well as medullary collecting ducts, with no appreciable staining in the loops of Henle (Figure 6H and K). 

## Discussion

In common with adult SHR [13], activation of UT by endogenous urotensin peptides has a greater impact upon renal haemodynamics in the young SHR with moderately elevated blood pressure compared with WKY rats. The pronounced natriuresis and diuresis that accompanied UT antagonism in the young SHR suggests that tonic activation of UT by urotensin peptides may contribute to the retention of sodium and water which precedes the rapid elevation in blood pressure and onset of hypertension as the SHR matures [16]. Differential renal urotensin peptide mRNA expression indicates that URP rather than UII is the major UT ligand in the young WKY rat and SHR. 

We have shown recently that the urotensin system is expressed in the fetal kidney from as early as embryonic day 19 (E19) and has not yet reached functional maturity by 4 weeks of age in the Sprague-Dawley (SD) rat [19]. When infused with either rUII (0.6-60 pmol. min^-1^. 100 g bwt^-1^) or URP (6 pmol. min^-1^. 100 g bwt^-1^), doses which evoke changes in renal function in the adult SD [2,5], young SD rats failed to respond. Young WKY rats were similarly unresponsive to rUII and URP infusion: the only measured variable which was significantly altered was ERBF. It should be noted that although GFR was significantly reduced following URP administration in young WKY rats, there were already differences in the baseline, untreated values between the three groups of animals. This reflects the variability of in vivo models and as such we are cautious in ascribing any biological significance to these data. In contrast to the modest changes seen in the young WKY animals, we have reported previously that adult WKY exhibit marked reductions in GFR and the excretion of water and sodium in response to rUII [13], implying that the urotensin system has a greater influence in the adult rat. 

Where the young SD and WKY rats differed was in their response to UT antagonism. Using the same antagonist, SB-706375, we observed marked increases in GFR, urine flow and sodium excretion rates in young SD rats [19], whereas young WKY rats in the current study responded in a more modest manner. Similar strain differences have been noted before with 70-80% lower binding of [^125^I]hUII to renal membranes [23] associated with 10-fold lower renal UT gene copy number in the adult WKY compared with the SD rat [2]. 

Like the young SD [19] and WKY rats, young SHR were relatively unresponsive to exogenous rUII infusion. URP induced a significant reduction in GFR; however urine flow and sodium excretion rates were unaltered in young SHR. In contrast adult SHR responded with robust reductions in GFR, urine flow and sodium excretion that exceeded the changes evoked by rUII in adult WKY rats [13]. It was not until the action of endogenous urotensin peptides was blocked by the administration of a UT antagonist that the magnitude of the influence of the urotensin system in the young SHR became apparent. UT antagonism resulted in 3-4 fold increases in urine flow and sodium excretion, driven by a marked increase in GFR in the young SHR. This response compares with 1.2-1.9 fold increases in the same parameters in adult SHR [13], suggesting that the urotensin system may play a greater role in the regulation of GFR and filtered load in young, pre-hypertensive SHR.

Adult SHR have higher renal expression levels of both UII and UT mRNA compared with WKY rats, with greater expression in the medulla than the cortex [13]. In contrast UII and UT mRNA expression did not differ between young SHR and WKY rats, nor was there a difference in expression levels between the cortex and medulla in the current study. Shi et al. [14] also reported that renal UII and UT mRNA expression were comparable between 5 week old SHR and WKY rats and that expression increased with age only in the SHR. Yet these authors also noted that renal tissue and plasma UII concentrations were higher in young SHR compared with both young WKY rats and adult rats of both strains. An explanation for this apparent inconsistency between UII mRNA expression and UII peptide concentration is offered by our current observation that URP mRNA expression is significantly higher in the young SHR compared with age-matched WKY rats. Available antibodies are unable to distinguish between UII and URP due to their close structural similarity; hence measurements of UII-immunoreactivity reflect both of these peptides. Furthermore, we have shown previously that URP mRNA expression is lower in adult SHR compared with WKY rats [2], implying that URP’s role in the regulation of renal function diminishes with increasing age in the SHR. Interestingly, the idea that UII and URP are able to bind to separate regions of UT leading to differing conformational changes and the activation of different intracellular signalling cascades has been proposed recently [24]. Consequently, up-regulation of URP in the young pre-hypertensive SHR may evoke UT-mediated effects that are not apparent in the adult SHR when URP expression falls and UII expression increases.

The kidney is known to play a major role in the development of hypertension in the SHR. Transplantation studies show that previously normotensive recipients of an SHR kidney develop hypertension [25]. Assessment of renal function in conscious young SHR reveals lower GFR [18], urine flow and sodium excretion rates compared with young WKY rats [16]. Thus the young pre-hypertensive rat undergoes a period of sodium and water retention which leads to an increase in blood pressure and the establishment of hypertension. We have demonstrated herein that acute administration of the UT antagonist SB-706375 resulted in marked increases in GFR, urine flow and sodium excretion rates in young SHR, implying that there is tonic regulation of renal filtration by the urotensin system in the young SHR. Although we have shown previously that UII has a direct effect on tubular sodium reabsorption [5], it seems unlikely that the anti-natriuretic action of endogenous URP/UII in the young SHR is mediated by a tubular effect. Excess sodium retention by the pre-hypertensive SHR has been localised to the proximal tubule [26]; however we observed UT expression only in the distal nephron in young SHR, reflecting our previous observations in adult SHR [13]. Hence, it seems more likely that URP/UII affected sodium handling by altering GFR and thus the filtered load of sodium entering the tubule.

One important point to note is that in our current study, which was conducted under non-recovery anaesthesia, baseline GFR measurements did not differ between young SHR and WKY rats. Using similar anaesthetic regimes, we [26] and others [27,28] have previously failed to replicate the difference in basal GFR reported in conscious SHR and WKY [18,29]. This is clearly a limitation to the current study; nonetheless the robust strain-specific response observed upon UT antagonism lends support to the proposal that endogenous urotensin peptides have a more profound influence on GFR in young pre-hypertensive SHR.

Another limitation of our study is that we were unable to administer the UT antagonist to young SHR chronically. Such an experiment is necessary to show whether inhibition of the urotensin system can prevent the rapid increase in systemic blood pressure seen in SHR at 6-8 weeks of age. Nonetheless, the current study has shown that urotensin system activity is increased in the young pre-hypertensive SHR. Evidence points towards URP rather than UII itself being the ligand that is up-regulated in the young SHR kidney. Acute inhibition of the urotensin system in the young SHR kidney increased GFR and thus the filtered load of sodium and water; variables which are known to be involved in the onset of hypertension in this model. Taken together, these data suggest that increased urotensin system activity may contribute to the onset of hypertension in the SHR.
